# Psychological Impact During the First Outbreak of COVID-19 on Frontline Health Care Workers in Shanghai

**DOI:** 10.3389/fpubh.2021.646780

**Published:** 2021-05-17

**Authors:** Jingjing Feng, Jinfu Xu, Susu Xu, Huifang Cao, Cuixia Zheng, Lokesh Sharma, Charles S. Dela Cruz, Jing Zhang, Dejie Chu, Li Yu, Chunlin Tu, Fan Li, Tao Ren, Fengying Zhang, Chunlin Du, Wenchao Gu, Hongwei Liu, Yechang Qian, Changxing Shen, Chunhong Tang, Yueping Bi, Feng Xiao, Kejia Gu, Jie Zhang, Zheng Ye, Liang Zhao, Jiayi Zhai, Xiaoying Hu, Jieming Qu, Zhijun Jie

**Affiliations:** ^1^Department of Pulmonary and Critical Care Medicine, Shanghai Fifth People's Hospital, Fudan University, Shanghai, China; ^2^Department of Respiratory and Critical Care Medicine, Shanghai Pulmonary Hospital, Tongji University School of Medicine, Shanghai, China; ^3^Department of Respiratory and Critical Care Medicine, Shanghai Fifth People's Hospital, Fudan University, Shanghai, China; ^4^Department of Respiratory Disease, Jing'an District Centre Hospital of Shanghai (Huashan Hospital Fudan University Jing'an Branch), Shanghai, China; ^5^Department of Respiratory and Critical Medicine, Yangpu Hospital, Tongji Universtiy, Shanghai, China; ^6^Section of Pulmonary, Critical Care and Sleep Medicine, Yale University School of Medicine, New Haven, CT, United States; ^7^Department of Pulmonary and Critical Care Medicine, Tongren Hospital, Shanghai Jiao Tong University School of Medicine, Shanghai, China; ^8^Department of Respiratory and Critical Care Medicine, Shanghai Eighth People's Hospital, Shanghai, China; ^9^Department of Pulmonary and Critical Care Medicine, Tongji Hospital, Tongji University School of Medicine, Shanghai, China; ^10^Department of Respiratory and Critical Medicine, Jiading Center Hospital, Shanghai University of Medicine & Health Sciences, Shanghai, China; ^11^Department of Respiratory and Critical Medicine, Songjiang Hospital Affiliated to Shanghai Jiao Tong University School of Medicine, Shanghai, China; ^12^Department of Pulmonary and Critical Care Medicine, Shanghai Sixth People's Hospital, Shanghai Jiao Tong University, Shanghai, China; ^13^Department of Respiratory Medicine, Shanghai Putuo District People' Hospital, Shanghai, China; ^14^Department of Respiratory Medicine, Zhongshan Hospital Subordinating Qingpu Hospital, Shanghai, China; ^15^Department of Respiratory Medicine, Shanghai Pudong New Area People's Hospital, Shanghai, China; ^16^Department of Respiratory Medicine, Fengxian Central Hospital, Shanghai, China; ^17^Department of Respiratory Disease, Baoshan District Hospital of Integrated Traditional Chinese and Western Medicine, Shanghai, China; ^18^Department of Respiratory and Critical Medicine, Shanghai Tenth People's Hospital, Shanghai, China; ^19^Gumei Community Health Service Center, Shanghai, China; ^20^Yinhang Community Health Service Center, Shanghai, China; ^21^Zhoujiaqiao Community Health Service Center, Shanghai, China; ^22^Jiuting Community Health Service Center, Shanghai, China; ^23^Nanqiao Community Health Service Center, Shanghai, China; ^24^Changfeng Community Health Service Center, Shanghai, China; ^25^Zhuanqiao Community Health Service Center, Shanghai, China; ^26^Jiading Town Community Health Service Center, Shanghai, China; ^27^Jiangchuan Community Health Service Center, Shanghai, China; ^28^Department of Pulmonary and Critical Care Medicine, Ruijin Hospital, Institutes of Respiratory Diseases, Shanghai Jiao Tong University School of Medicine, Shanghai, China

**Keywords:** COVID-19 pandemic, hospital care workers, psychological distress, first outbreak, GHQ-12

## Abstract

**Background:** The COVID-19 pandemic is a significant health threat. Health care worker (HCWs) are at a significant risk of infection which may cause high levels of psychological distress. The aim of this study was to investigate the psychological impact of the COVID-19 on HCWs and factors which were associated with these stresses during the first outbreak in Shanghai.

**Methods:** Between February 9 and 21, 2020, a total of 3,114 frontline HCWs from 26 hospitals in Shanghai completed an online survey. The questionnaire included questions on their sociodemographic characteristics, 15 stress-related questions, and General Health Questionnaire-12 (GHQ-12). Exploratory factor analysis was applied to the 15 stress-related questions which produced four distinct factors for evaluation. Multiple linear regression models were performed to explore the association of personal characteristics with each score of the four factors. Binary logistic analysis was used to explain the association of personal characteristics and these four factors with the GHQ-12.

**Results:** There were 2,691 valid surveys received. The prevalence of emotional distress (defined as GHQ-12 ≥ 12) was noted in 47.7% (95%CI:45.7–49.6%) HCWs. Females (OR = 1.43, 95%CI:1.09–1.86) were more likely to have a psychological distress than males. However, HCWs who work in secondary hospitals (OR = 0.71, 95% CI:0.58–0.87) or had a no contact history (OR = 0.45, 95%CI: 0.35–0.58) were less likely to suffer psychological distress. HCWs who were nurses, married, and had a known contact history were highly likely to have anxiety. HCWs working at tertiary hospitals felt an elevated anxiety regarding the infection, a lack of knowledge, and less protected compared to those who worked at secondary hospitals.

**Conclusions:** Our study shows that the frontline HCWs had a significant psychosocial distress during the COVID-19 outbreak in Shanghai. HCWs felt a lack of knowledge and had feelings of being not protected. It is necessary for hospitals and governments to provide additional trainings and psychological counseling to support the first-line HCWs.

## Introduction

The novel coronavirus disease 2019 (COVID-19) emerged as a major healthcare challenge which has spread across the world. The etiological agent for COVID-19 was identified as an enveloped RNA betacoronavirus (1) that is named severe acute respiratory syndrome coronavirus 2 (SARS-CoV-2), which has a phylogenetic similarity to SARS-CoV (2). Despite 1 year having passed since its emergence, COVID-19 continues to cause extensive disease and death without any effective treatment. Healthcare professionals have been at major risks of epidemic especially at the first outbreak of COVID-19. It has infected at least 1,716 healthcare workers including six fatalities by February 14, 2020 (3), which was at the beginning of the epidemic in China.

World Health Organization (WHO) declared the COVID-19 as a public health emergency of international concerns (4). The pandemic had created considerable anxiety and panic worldwide including in China (5, 6). Health Care Workers (HCWs) encountered an increasing workload and a perception of being at an increased risk of infection, as in any infectious disease outbreaks. These conditions could possibly affect their psychological well-being. It is expected that the current epidemic has placed significant stresses on people including HCWs, especially during the first outbreak when there was limited knowedge regarding its transmission, disease course, and pathogenesis (7). A lot of studies conducted in China and other countries had reported that HCWs suffers depression, anxiety, and stress because of COVID-19 (8–11). Previous studies has shown that many of the HCWs presented high levels of psychological distress during these outbreaks such as the severe acute respiratory syndrome (SARS) (12), H1N1 (13), and H7N9 (14) outbreaks.

Several studies have demonstrated the psychological impact of the epidemic on health care workers in different aspects, such as medical staff working in different risk workplace (15), different medical care population in front of this pandemic (16, 17), and posttraumatic stress disorder symptoms (PTSD) (18, 19). However, only a few studies have looked into the psychological stress during the first outbreak outside Wuhan, when a very limited knowledge of the disease existed. The lack of knowledge regarding COVID-19 and absence of any effective medicine or vaccine at that time made HCWs highly vulnerable to stress. Due to the high risk of exposure, the medical staff is at a high risk of SARS-CoV-2 virus infection.

Shanghai is the largest city in China with a population of ~24 million. The first case of COVID-19 was identified on January 20, 2020 in Shanghai. A total of 342 cases was confirmed until March 8, 2020, which were scattered throughout the city (20). At the meantime, Wuhan was in the center of the pandemic. More than 40,000 medical staff from other provinces including Shanghai has been deployed to the Hubei province to assist with the medical needs in Wuhan since January 25, 2020 (15). At the beginning of the outbreak, the lack of knowledge and rising cases of death induced high levels of stress to the public. We sought to investigate the psychological impact of the initial COVID-19 outbreak in these frontline HCWs, who are working in the departments of respiratory, emergency, or infectious diseases. There were 975 physicians and 1,584 nurses who completed the survey from 26 hospitals. Twelve item version of General Health Questionnaire (GHQ-12) survey was used as described previously (21). The GHQ-12 is a well-established method to quantify stress and has been used in a wide range of conditions (22, 23). Our questionnaire was designed to assess four major factors based on the relevant factors during the early stage of pandemic. These factors included anxiety related to the infection, awareness of the COVID-19, feeling of being protected from infection, and attitude toward work in the face of the current outbreak, as described before (24, 25). Although studies had reported that COVID-19 had caused psychological problems around the world, this is the first study to define the stress in HCWs at the early stage of the pandemic in Shanghai, when limited knowledge about COVID-19 existed.

Our data showed that there was a significant psychological distress among the HCWs in Shanghai hospitals. The psychological distress was higher among the HCWs who were working in tertiary hospitals, females, and unsure of their contact history with COVID-19 infected subjects.

## Methods

### Subjects and Procedure

HCWs from 26 hospitals in 14 out of the 16 districts in Shanghai were invited to participate in this survey. The two excluded districts are the Huangpu and Chongming districts. Huangpu has a fewer hospitals than others due to the small area, while Chongming is an island far away from the center of Shanghai. The data for this study were collected between February 9 and February 21, 2020, which was approximately around the peak of the COVID-19 outbreak in Shanghai. The questionnaire was completed through an online survey platform (“SurveyStar,” Changsha Ranxing Science and Technology, Shanghai, China). A total of 3,114 frontline HCWs, from seven tertiary, 10 secondary, and nine primary hospitals (referred as community health centers in China) were invited to participate. Three types of hospitals are organized according to a three-tier system in China (26). Two thousand six hundred ninety-one (2,691) of the potential participants returned the survey (86.4% return rate). Among these responses, 132 were excluded because they failed to answer the quality check question correctly which explicitly asked them to pick the last option. A total of 2,559 surveys were analyzed.

### Content of the Questionnaire

This questionnaire consisted of three sections: sociodemographic characteristics, GHQ-12, and stress-related questions associated with COVID-19. The sociodemographic characteristics included gender, age, occupation, technical title, marital status, level of hospital, and contact history with suspected or confirmed cases.

GHQ-12 was used to assess the HCWs' psychological distress. GHQ-12 is a well-standardized measure of recent emotional distress, which is the most widely used tool in quantitative social science and epidemiology for the analysis of mental health trends (21, 27, 28). Studies have shown the usefulness of GHQ-12 to assess the psychological impact of SARS among HCWs (21). A 4-point Likert scoring method (0, never; 1, occasionally; 2, often; 3, almost always) was used in this study. A threshold score of >12 was used to identify the presence of emotional distress (27).

To assess the HCWs' concerns and worries about the pandemic, knowledge about COVID-19, their attitude toward work during the pandemic, and whether these factors were associated with personal characteristics, we designed 15 items of stress-related questions (**Table 2**). These questions were based on the previous studies of similar novel infection mediated outbreaks including the SARS (29), H1N1 pandemic (24), and avian influenza (30). We used a factor analysis to classify these 15 items to four distinct aspects of psychological stress. (a) “Anxiety about infection” included five items about the disease's dangerousness, perception of personal risk, perception of being a risk to family or friends, isolation from family, and functional ability toward social relationship. (b)“Knowledge about COVID-19” included questions about the epidemiology, symptoms, prognosis, and treatment. (c)“Feeling of being protected” included questions about the perception of feeling safe during work and at home. (d)“Attitude toward work” is designed to determine the willingness of HCWs to move to a position involving a high risk of contacting infection including moving to the epicenter of the disease. Each item was scored from 1 to 5, representing not at all, barely, a little, high, and very high, respectively. The survey was approved by the Ethical Review Board (2020-046) and the participation was voluntary.

### Statistical Analysis

Summary statistics for all variables and the prevalence of psychological distress were calculated. The data are expressed as mean ± standard deviation (SD) or as a number (percent). We applied a confirmatory factor analysis to the 15 stress-related questions. Principal component analysis with varimax rotation was used for factor analysis. For each factor, the total score of the stress-related questions was calculated. Multiple linear regression models were performed to explore the association of personal characteristics with each score of the four factors. Binary logistic analyses were used to explain the association of personal characteristics and these four factors with GHQ-12.

Statistical analysis were carried out using the SPSS (version 21) and graphs were prepared using the GraphPad Prism 8.

## Results

### Demographic Information

The basic demographic information was given in Table 1. A total of 2,559 answers from 26 hospitals in 14 out of the 16 districts of Shanghai were analyzed. The participants included 975 physicians (38.1%) and 1,584 nurses (61.9%). Majority of the responders were female, with a proportion of 68.8% in physicians and 97.9% in nurses, which reflects the overall population of the HCWs in Shanghai hospitals. Most of the nurses were 26–35 years old (47.6%) while most of the physicians were 36–45 years old (45.7%). Most of the HCWs were married. HCWs with junior and middle levels of technical title consisted the majority of the participants in this survey. 34.6 and 37.8% of participants in physicians were from secondary hospitals and community healthcare center, respectively. 48.9% of the nurses were from a secondary hospital. Most of them, 55.5% of the physicians and 67.8% of the nurses, did not had a suspected or confirmed contact with COVID-19 patients.

**Table 1:** The characteristic of HCWs.

**Characteristics**	**Physicians**	**Nurses**
	**(*n* = 975)**	**(*n* = 1,584)**
**Gender**		
Male	304 (31.2)	33 (2.1)
Female	671 (68.8)	1,551 (97.9)
**Age**		
Under 25	13 (1.3)	277 (17.5)
26–35	296 (30.4)	754 (47.6)
36–45	446 (45.7)	359 (22.7)
Up to 46	220 (22.6)	194 (12.2)
**Marriage**		
Married	821 (84.2)	1,050 (66.3)
Single	131 (13.4)	495 (31.3)
Divorce	23 (2.4)	39 (2.5)
**Title**		
Junior	203 (20.8)	1,135 (71.7)
Middle	559 (57.3)	443 (28)
Senior	213 (21.8)	6 (0.4)
**Level of hospital**		
Tertiary	269 (27.6)	431 (27.2)
Secondary	337 (34.6)	774 (48.9)
Community	369 (37.8)	379 (23.9)
**Contract history**		
Yes	194 (19.9)	205 (12.9)
No	541 (55.5)	1,074 (67.8)
Not sure	240 (24.6)	305 (19.3)

### Factors Associated With the Presence of Psychological Distress

**Figure 1:**
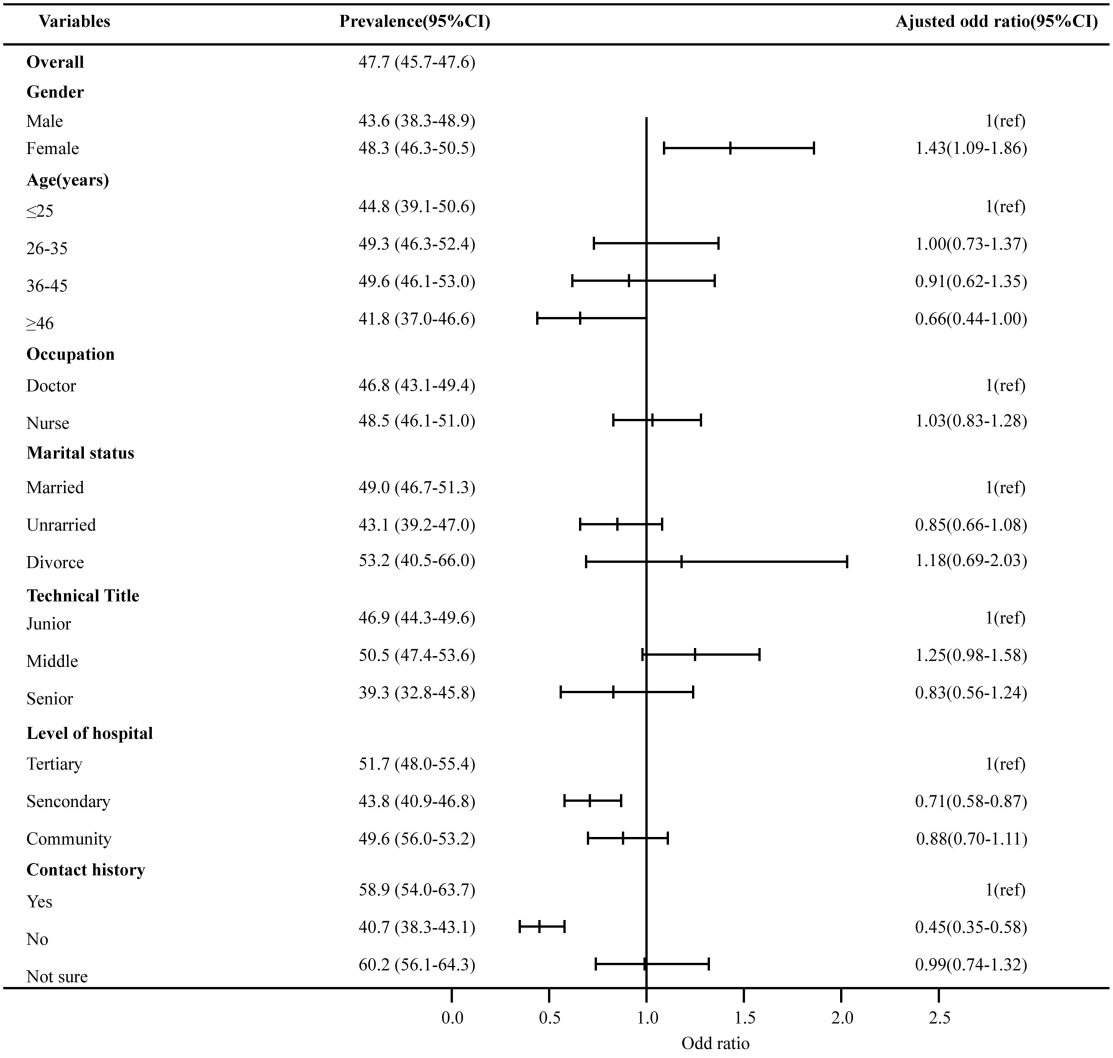
Factors associated with the presence of psychological distress. The prevalence of psychological distress with 95% CI of independent variables is presented on the left column. Multivariate analyses were used to calculate the adjusted odds ratio of psychological distress, adjusted ORs, and their 95% CI were shown in the figures, and the numbers are given in the right column.

The Cronbach alpha coefficient of the GHQ-12 in this study was 0.81, a level considered good for internal consistency. The prevalence of high scores (GHQ-12 ≥ 12) was in 47.7% (95%CI 45.7–49.6%) of the respondents, which indicated an elevated psychological distress among HCWs. As shown in Figure 1, multivariate analyses found that females were more likely to have a higher emotional distress than males (OR = 1.43, 95%CI: 1.09–1.86). However, HCWs working at secondary hospitals (OR = 0.71, 95%CI: 0.58–0.87) or among those who had a no known contact history (OR = 0.45, 95%CI: 0.35–0.58) were less likely to have a psychological distress compared with those who are working at tertiary or primary hospitals or had a known or suspected contact history. In contrast, age, marital status, occupation, and technical title were not associated with psychological distress.

### The Association of Personal Characteristics With Each Score of the Four Factors

Four factors were classification from the 15 questionnaire items having factor loadings ≥ 0.40 (Table 2). Exploratory factor analysis on the 15 items of perceived threat yielded four factors (explaining 68.8% of the total variance; KMO = 0.83). The Cronbach alpha coefficient of the 15 stress-related questions was 0.80, again indicating a good internal consistency and reliability. Estimated associations between the sociodemographic characteristics with the total score in each of the four factors is indicated in Table 3. The detailed scores for the four factors were showed in Supplementary Table 1. The comparison between nurse and physician in the four factors was also analyzed (Please see Supplementary Table 2).

**Table 2:** Factor analysis of the 15 stress-related questions.

**Questions**	**F1**	**F2**	**F3**	**F4**
**Factor 1: Anxiety about infection (Cronbach** ***α*** **= 0.82)**				
The disease's dangerousness	**0.476**	0.031	−0.058	−0.025
Anxiety about being infected	**0.870**	−0.064	−0.286	−0.176
The risk for family and relatives' to be infected	**0.851**	−0.043	−0.279	−0.174
Isolation from family and/or social environment	**0.737**	−0.107	−0.249	−0.223
The consequences on your functional ability regarding family, work, or social relationships	**0.794**	−0.055	−0.235	−0.113
**Factor 2: Knowledge about COVID-19 (Cronbach** ***α*** **= 0.89)**				
I believe that I have learned enough knowledge about epidemiology	−0.054	**0.826**	0.275	0.198
I believe that I have learned enough knowledge about symptom	−0.045	**0.892**	0.323	0.230
I believe that I have learned enough knowledge about prognosis	−0.086	**0.860**	0.307	0.221
I believe that I have learned enough knowledge about treatment	−0.056	**0.833**	0.332	0.256
**Factor 3: Feeling of being protected (Cronbach** ***α*** **= 0.87)**				
Feeling of being protected during work	−0.297	0.342	**0.863**	0.392
Feeling of being protected by local government	−0.194	0.310	**0.886**	0.289
Feeling of being protected by environment	−0.321	0.314	**0.912**	0.355
**Factor 4: Attitude toward work (Cronbach** ***α*** **= 0.77)**				
If you are asked to Wuhan, you will go without any hesitation	−0.200	0.199	0.304	**0.868**
If you are asked to change your position, which has more chance to contract COVID-19 patients, do you totally agree	−0.201	0.219	0.349	**0.870**
You think COVID-19 is a good chance to improve your clinical ability	−0.096	0.250	0.321	**0.742**

*Bold, factor loading ≥ 0.40*.

**Table 3:** The association of personal characteristics with each score of the four factors.

	**Factor 1^†^: Anxiety about infection**	**β**	***P***	**Factor 2^‡^: Knowledge about COVID-19**	**β**	***P***	**Factor 3^§^: Feeling of being protected**	**β**	**P**	**Factor 4^¶^: Attitude toward work**	**β**	***P***
	**Total score**			**Total score**			**Total score**			**Total score**		
	**(mean ± SD)**			**(mean ± SD)**			**(mean ± SD)**			**(mean ± SD)**		
**Gender**												
Male	14.11 ± 3.56			17.00 ± 2.47			10.32 ± 2.42			11.40 ± 2.65		
Female	14.19 ± 3.67	−0.02	0.339	16.55 ± 2.66	−0.054	**0.011**	10.50 ± 2.51	0.049	**0.018**	11.27 ± 2.73	−0.052	**0.016**
**Age (years)**												
≤ 25	13.58 ± 3.63			16.11 ± 2.74			11.29 ± 2.42			11.84 ± 2.57		
26–35	14.38 ± 3.71	0.049	0.178	16.55 ± 2.68	0.025	0.498	10.45 ± 2.56	−0.11	**0.003**	11.06 ± 2.75	−0.141	**<0.001**
36–45	14.30 ± 3.58	0.027	0.528	16.81 ± 2.58	0.014	0.749	10.14 ± 2.42	−0.104	**0.014**	11.21 ± 2.68	−0.137	**0.002**
≥46	13.87 ± 3.63	−0.014	0.692	16.72 ± 2.54	−0.011	0.763	10.64 ± 2.39	−0.014	0.706	11.60 ± 2.75	−0.073	**0.05**
**Occupation**												
Physician	13.81 ± 3.41			16.72 ± 2.50			10.00 ± 2.36			11.09 ± 2.73		
Nurse	14.41 ± 3.78	0.123	**<0.001**	16.54 ± 2.71	0.027	0.285	10.78 ± 2.54	0.12	**<0.001**	11.40 ± 2.71	0.115	**<0.001**
**Marital status**												
Married	14.35 ± 3.69			16.75 ± 2.59			10.37 ± 2.52			11.26 ± 2.74		
Unmarried	13.61 ± 3.46	−0.103	**<0.001**	16.16 ± 2.75	−0.084	**0.001**	10.85 ± 2.41	0.002	0.95	11.35 ± 2.66	0.014	0.575
Divorce	14.92 ± 3.91	0.024	0.217	16.90 ± 2.62	0.008	0.687	10.21 ± 2.40	−0.016	0.405	11.35 ± 2.79	0.001	0.945
**Technical title**												
Junior	14.20 ± 3.72			16.40 ± 2.73			10.75 ± 2.55			11.23 ± 2.71		
Middle	14.32 ± 3.64	0.018	0.513	16.79 ± 2.52	0.061	**0.028**	10.18 ± 2.46	−0.031	0.252	11.24 ± 2.73	0.066	**0.016**
Senior	13.45 ± 3.28	−0.037	0.153	17.02 ± 2.49	0.067	**0.011**	10.20 ± 2.18	−0.005	0.841	11.81 ± 2.67	0.114	**<0.001**
**Level of Hospital**												
Tertiary	14.92 ± 3.69			16.28 ± 2.66			10.23 ± 2.55			11.12 ± 2.85		
Secondary	13.88 ± 3.57	−0.122	**<0.001**	16.92 ± 2.62	0.111	**<0.001**	10.89 ± 2.48	0.094	**<0.001**	11.42 ± 2.68	0.035	0.137
Primary	13.94 ± 3.67	−0.109	**<0.001**	16.46 ± 2.59	0.011	0.636	10.11 ± 2.40	−0.002	0.929	11.23 ± 2.64	0.03	0.212
**Contact history**												
Yes	15.01 ± 3.94			16.87 ± 2.54			10.41 ± 2.34			11.35 ± 2.78		
No	13.60 ± 3.43	−0.187	**<0.001**	16.74 ± 2.62	0.002	0.949	10.85 ± 2.44	0.072	**0.007**	11.47 ± 2.67	0.017	0.527
Not sure	15.30 ± 3.72	0.029	0.272	16.03 ± 2.70	−0.108	**<0.001**	9.43 ± 2.49	−0.15	**<0.001**	10.69 ± 2.74	−0.01	**<0.001**

*Adjusted for gender, age, occupation, technical title, marital status, level of hospital, and Contact history*.

† *R^2^ = 0.083, adjusted R^2^ = 0.078*.

‡ *R^2^ = 0.041, adjusted R^2^ = 0.036*.

§ *R^2^ = 0.091, adjusted R^2^ = 0.087*.

¶ *R^2^ = 0.036, adjusted R^2^ = 0.031*.

*^β^, standardized partial regression coefficient*.

*The bold values indicates p < 0.05*.

#### Worries About the COVID-19 Pandemic

For factor 1, “Anxiety about infection,” nurses were more likely to have anxiety than physicians [partial regression coefficient (B) = 0.926, SE = 0.178, β = 0.123, *P* < 0.001]. Married or divorce workers were associated with more anxiety than unmarried workers (B = −0.874, SE = 0.214, β = −0.103, *P* < 0.001). HCWs at tertiary hospitals felt more anxiety than those at secondary or primary hospitals (secondary hospitals: B = −0.901, SE = 0.171, β = −0.122, *P* < 0.001; primary hospitals: B = −0.875, SE = 0.191, β = −0.109, *P* < 0.001). In regard to contact history, people who had a no known contact history felt less anxiety compared to those who had a known contact with a COVID-19 patient or were not sure about their contact history (B = −1.413, SE = 0.202, β = −0.187, *P* < 0.001).

#### Perceived Knowledge of the COVID-19 Pandemic

For factor 2, “Knowledge about COVID-19,” females felt that they were less aware about the COVID-19 than their male counterparts (B = −0.424, SE = 0.168, β = −0.054, *P* = 0.011). Unmarried HCWs reported less knowledge compared to the married and divorced HCWs (B = −0.518, SE = 0.158, β = −0.084, *P* = 0.001). HCWs at tertiary hospitals were more likely to feel that they had less knowledge compared to HCWs at secondary hospitals (B = 0.59, SE = 0.126, β = 0.111, *P* < 0.001). In regard to the contact history, HCWs who were not sure of their contact history felt that they lack knowledge more (B = −0.698, SE = 0.174, β = −0.108, *P* < 0.001). There was no association between the age, occupation, and technical title and perception of knowledge about COVID-19.

#### Concerns on Environmental Safety During the COVID-19 Pandemic

For factor 3, “Feeling of being protected,” males felt less protected than the female HCWs (B = 0.364, SE = 0.154, β = 0.049, *P* = 0.018). Similarly, physicians also thought that they lacked protection than the nurses (B = 0.617, SE = 0.127, β = 0.12, *P* < 0.001). HCWs who were 25–35 y and 36–45 y felt less protected (25–35y: B = −0.558, SE = 0.185, β = −0.11, *P* = 0.003; 36–45y: B = −0.56, SE = 0.227, β = −0.104, *P* = 0.014). Workers at secondary hospitals thought they were well-protected compared with workers at tertiary hospitals and community service centers (B = −0.475, SE = 0.117, β = 0.094, *P* < 0.001). In regard to the contact history, workers who did not have a contact history felt well-protected than others (B = 0.371, SE = 0.137, β = 0.072, *P* = 0.007). On the other hand, HCWs who were not sure of their contact history felt less protected (B = −0.917, SE = 0.16, β = −0.15, *P* < 0.001).

#### Impact on Work Attitude During the COVID-19 Pandemic

For factor 4, “Attitude toward work,” males and nurses had a more positive attitude toward work than females (B = −0.416, SE = 0.173, β = −0.052, *P* = 0.016) and physicians (B = 0.646, SE = 0.142, β = 0.115, *P* < 0.001), respectively. HCWs of age under 25 y were the most willing group of all ages when they were asked to move to a position involving a high risk of contacting infection. HCWs with a technical title of senior showed a positive attitude toward work than HCWs with junior or middle titles (B = 1.111, SE = 0.258, β = 0.114, *P* < 0.001). HCWs who were not sure of their contact history had a negative attitude toward work (B = −0.665, SE = 0.18, β = −0.01, *P* < 0.001).

### The Association of GHQ-12 With Scores of Each of the Four Factors

Logistic regression was performed to identify the association between emotional distress and the four factors as shown in Table 4. All four factors were positively correlated with a higher distress. The feeling of anxiety about infection was positively correlated with a high GHQ-12 score [OR = 1.518 (1.467, 1.571)]. Both the feeling of a lack of knowledge and being less protected led to a higher anxiety and psychological distress [OR = 0.895 (0.868, 0.923) and OR = 0.74 (0.713, 0.768), respectively]. HCWs with a positive attitude toward work had less psychological distress [OR = 0.839 (0.814, 0.865)]. Furthermore, the correlation among these factors are indicated in Supplementary Figure 1.

**Table 4:** Binary logistic analysis of the four factors with a positive GHQ-12.

**Factors**	**β**	**SE**	**OR**	**95%CI**	***P*-value**
Factor 1					
Anxiety about infection	0.418	0.017	1.518	1.467–1.571	<0.001
Factor 2					
Knowledge about COVID-19	−0.111	0.016	0.895	0.868–0.923	<0.001
Factor 3					
Feeling of being protected	−0.301	0.019	0.74	0.713–0.768	<0.001
Factor 4					
Attitude toward work	−0.175	0.015	0.839	0.814–0.865	<0.001

## Discussion

The ongoing pandemic of COVID-19 is one of the worst respiratory viral infections in the twenty-first century. It has surpassed the SARS (8,098 patients with 774 deaths) outbreak of 2002–2003 (31) and MERS outbreak (2,506 infections with 862 deaths) of 2012 (32). Due to the highly contagious nature of the infection, HCWs are at a heightened risk of getting the coronavirus infection (33). At the first few weeks of this pandemic, a sharp increase in the number of confirmed cases, along with the lack of a clear knowledge about the possible modes of transmission and availability of effective therapeutics, has created widespread panic in the general population, including among the HCWs. At the same time, HCWs from all over the country were asked to volunteer to go to Wuhan to serve at the epicenter of the disease. The volunteers included HCWs from Shanghai, especially those working in the departments of Respiratory, Emergency, and Infectious diseases. Although many studies have reported the psychological effects during COVID-19, a few studies have focused on the psychologic problems experienced by HCWs in Shanghai and during the early stage of the pandemic.

Some studies have reported that COVID-19 caused psychological impacts in other countries as the pandemic has spread all over the world. The study from Italy has shown that the greatest prevalence of psychological distress was reported in the <34 years age group and in north Italy. The psychological impact influenced important daily life activities, such as sexuality and nutrition among the general population (34). Leivy Patricia et al. reported that during the phase 2 of the COVID-19 outbreak in Mexico, psychological distress and post-traumatic stress symptoms were present in over a quarter of the population (35). A French study reported that a hospital staff displayed the psychological consequences of pandemic stress, resulting in the use of anxiolytics and sleeping pills (36). Another survey on nurses in the USA showed that nurses who lack access to adequate personal protective equipment were more likely to report symptoms of depression, anxiety, and post-traumatic stress disorder (37).

Our study shows that 47.7% of the HCWs experienced psychological distress based on the GHQ-12 score. We only chose the GHQ-12 which has been used in various studies for many years because of the intensive schedule of the medical staff. Fu et al. used the GHQ-12 to study the psychological impact of the COVID-19 cases on the medical staff of Beijing Xiaotangshan Hospital on March 20 to 29, 2020. The results show that the medical staff working at Xiaotangshan Hospital underwent relatively low levels (17%) of emotional distress (38). A meta-analysis of other studies showed that the prevalence of anxiety in healthcare workers was 26% (18–34%), ranged between 7% (5–9%) in Singapore and 57% (52–63%) in Italy (9). Other studies which looked into PTSD reported that 40.2% of the health care professionals indicated positive screens for significant posttraumatic stress disorder symptoms (18). To investigate further in-depth of the mental state of HCWs, a French study reported that 32% had symptoms of anxiety, 16% of depression, and 16% of post-traumatic stress disorder (36). Compared to these studies on the psychological impact of COVID-19 among health care workers, our study showed a higher prevalence of psychological distress in HCWs. This difference may be explained by the following reasons: Firstly, our study was conducted between February 9 to 21, 2020, which was the beginning of the outbreak, while other studies were carried out at the later stages. Secondly, other studies, including in China for the dedicated COVID-19 hospitals such as the Beijing Xiaotangshan Hospital, reported a lower psychological stress (38). This may be due to the renovation of the Beijing Xiaotangshan Hospital as the designated hospital for the screening and treating of COVID-19 cases, which had sufficient medical and psychological preparations. Compared with other studies on the psychological impact of COVID-19 among health care workers using other measurements, our study used the GHQ-12. This was simpler than other studies who used more than one scale, such as the IES (Impact of Event Scale), Seven-Item Generalized Anxiety Disorder Scale (GAD-7), and DASS (Depression, Anxiety, and Stress Scale). Different measurement tools may bring different results. Lack of knowledge and insufficient psychological coping strategies to the disease at the first outbreak may be essential components to make an impact on the psychological well-being.

However, other studies also revealed a prevalence of psychological problems, which was even higher than our study. Marques used the GHQ-12 to study the impact of COVID-19 on the psychological health of university students in Spain. The result shows that 52.1% of the respondents were classified as high scores (39). Another study demonstrated a high prevalence of mental health symptoms among health care workers treating patients with COVID-19 in China, which revealed that 50.4, 44.6, 34.0, and 71.5% of all the participants reported symptoms of depression, anxiety, insomnia, and distress, respectively (8). Reports of the psychological impact of SARS on hospital staff indicated that high levels of distress were common (40). During the SARS epidemics, 68% of the HCWs reported a high level of stress and about 57% were found to have experienced psychological distress in Hong Kong (25).

In this study, HCWs who were female, working at tertiary hospitals, with confirmed or unsure contact history had a significantly elevated psychological distress. However, the level of stress was not significantly affected by age, occupation, technical total, and marital status. Females more easily perceive a higher lack of knowledge about COVID-19 than males in our study, a possible contributing factor to distress in females. A significant association was noted between the prevalence of psychological distress and contact history with suspected or confirmed patients. HCWs who had a no known contact history had a lower psychological distress compared to those who had a known contact with a COVID-19 patient or were unsure of their contact history. On similar lines, Ko et al. (41) also reported that a direct or indirect exposure to SARS would bring a greater psychological impact on the public. Tang et al. (14) also showed that the PTSD level among physicians and nurses after their exposure to H7N9 patients was high. Nurses were more likely to have a higher distress about being infected compared to the physicians. Similar results were reported in other studies about the COVID-19 (8, 42) and SARS outbreak (31, 43). Nurses often need to spend more time with infectious patients and are in closer contact with patients. Nurses constitute the largest workforce in the hospitals and are directly and intensively involved in patient care, experiencing a greater risk.

Higher number of patients including the number of beds in tertiary hospitals may increase the chances of contact with suspected or confirmed patients. On the other hand, many HCWs were asked to volunteer to Wuhan after the out coming traffic from Wuhan was blocked on January 23, 2020. Most of these HCWs going to Wuhan came from tertiary hospitals. This may be one of the contributing factors for a higher psychological distress in tertiary hospitals. Factor analysis results show that HCWs at tertiary hospitals and people unsure of their contact history had a higher anxiety about the infection, felt that they lacked the knowledge, and felt less protected than those at secondary hospitals and people with no contact history, indicating that all the three factors might contribute to psychological distress. HCWs working in primary hospitals were more likely to have a higher emotional distress than those at secondary hospitals. At the early stage of the pandemic, many of the suspected patients from other cities and community residents were recommended to isolate themselves at home or hotels for 14 days. The medical staffs at primary hospitals were responsible for monitoring their health changes. This may have led to the higher distress in these HCWs.

The results revealed that HCWs who had a higher anxiety regarding the infection were more likely to have a psychological distress. The feeling of lack of knowledge and not being protected led to higher levels of anxiety. These factors emphasize the need to minimize the stress at work. Decreasing psychological distress could give HCWs a positive attitude toward work when they are needed at the frontline during emerging pandemics such as COVID-19.

Our study was conducted during the peak of the initial phase of the COVID-19 outbreak in Shanghai, when information was changing rapidly and knowledge about the disease was limited. As the disease evolves and institutional policies are implemented, the HCWs' perceptions and experience may change. Future follow-up investigations, using both qualitative and quantitative approaches, will be necessary to understand the psychosocial effects of COVID-19 on HCWs over time after significant knowledge and preventive vaccines are administered. Because the adjusted *R*^2^ in Table 3 is low, future studies should look for other variables that can better explain the psychological impact of a pandemic. Overall, we recommend an appropriate training and support to the HCWs to ensure their psychological well-being, as effective clinical service depends on their overall and psychological well-being.

### Limitations

This study has some limitations. The multicenter design of the study did not allow us to transpose its conclusions to all types of hospital staff. Most of the respondents were female which may limit the generalizability of the findings. Another limitation is that only one measurement tool was used to evaluate the HCWs' psychological problems, which might be insufficient to evaluate all psychological problems. Thirdly, we did not evaluate the psychological distress of the participants after the pandemic. A follow-up study on the same participants should be conducted to observe the persistence of the perceived psychological distress.

## Conclusion

This study examined the emotional distress experienced by HCWs responding to COVID-19 in Shanghai, China during the first outbreak of the disease outside Wuhan. HCWs felt a lack of knowledge about COVID-19 and had feelings of being unprotected. It is necessary for hospitals and governments to implement not only more prevention strategies, but also psychological supports for the frontline HCWs.

## Data Availability Statement

The original contributions presented in the study are included in the article/Supplementary Material, further inquiries can be directed to the corresponding author/s.

## Ethics Statement

The studies involving human participants were reviewed and approved by Shanghai Ethical Review Board (2020-046) and the participation was voluntary. Written informed consent for participation was not required for this study in accordance with the national legislation and the institutional requirements.

## Author Contributions

JF for interpretation of the data, preparation, funding acquisition, and writing-original draft. JX for conceptualization and design. SX for software, writing-review, and editing. LS and CSD for writing-review and editing. HC, CZ, JinZ, DC, LY, CTu, FL, TR, FZ, CD, WG, HL, YQ, CS, CTa, YB, FX, KG, JieZ, ZY, LZ, JZhai, and XH for resources supply. JQ for conceptualization, writing-review, and editing. ZJ for conceptualization, interpretation of the data, resources, funding acquisition, writing-review, and editing. All authors contributed to the article and approved the submitted version.

## Conflict of Interest

The authors declare that the research was conducted in the absence of any commercial or financial relationships that could be construed as a potential conflict of interest.

## Supplementary Material

Supplementary Figure 1 Correlation among the four factors: Spearman correlation analysis was performed to assess the correlation among the four factors. In total, six figures were shown to express correlations between each other. Spearman *r*-value and *p*-value are shown beside each figure.

Supplementary Table 1 The score of the four factors with personal characteristics.

Supplementary Table 2 Factors and prevalence of psychological problem in different occupation.

## Abbreviations

HCWs: Health care worker
GHQ-12: General Health Questionnaire-12
COVID-19: The novel coronavirus disease 2019
WHO: World Health Organization
SARS: severe acute respiratory syndrome
MERS: Middle East Respiratory Syndrome Coronavirus
Cis: Confidence intervals
β: Standardized partial regression coefficients
PTSD: Post-traumatic stress disorder
IES: Impact of Event Scale
GAD-7: Seven-Item Generalized Anxiety Disorder Scale
DASS: Depression, Anxiety and Stress Scale.
