# Supplementary figures and images for: Psychological Impact During the First Outbreak of COVID-19 on Frontline Health Care Workers in Shanghai
Source: Front Public Health. 2021 May 17;9:646780. doi: 10.3389/fpubh.2021.646780 (PMC8165161; doi:10.3389/fpubh.2021.646780)

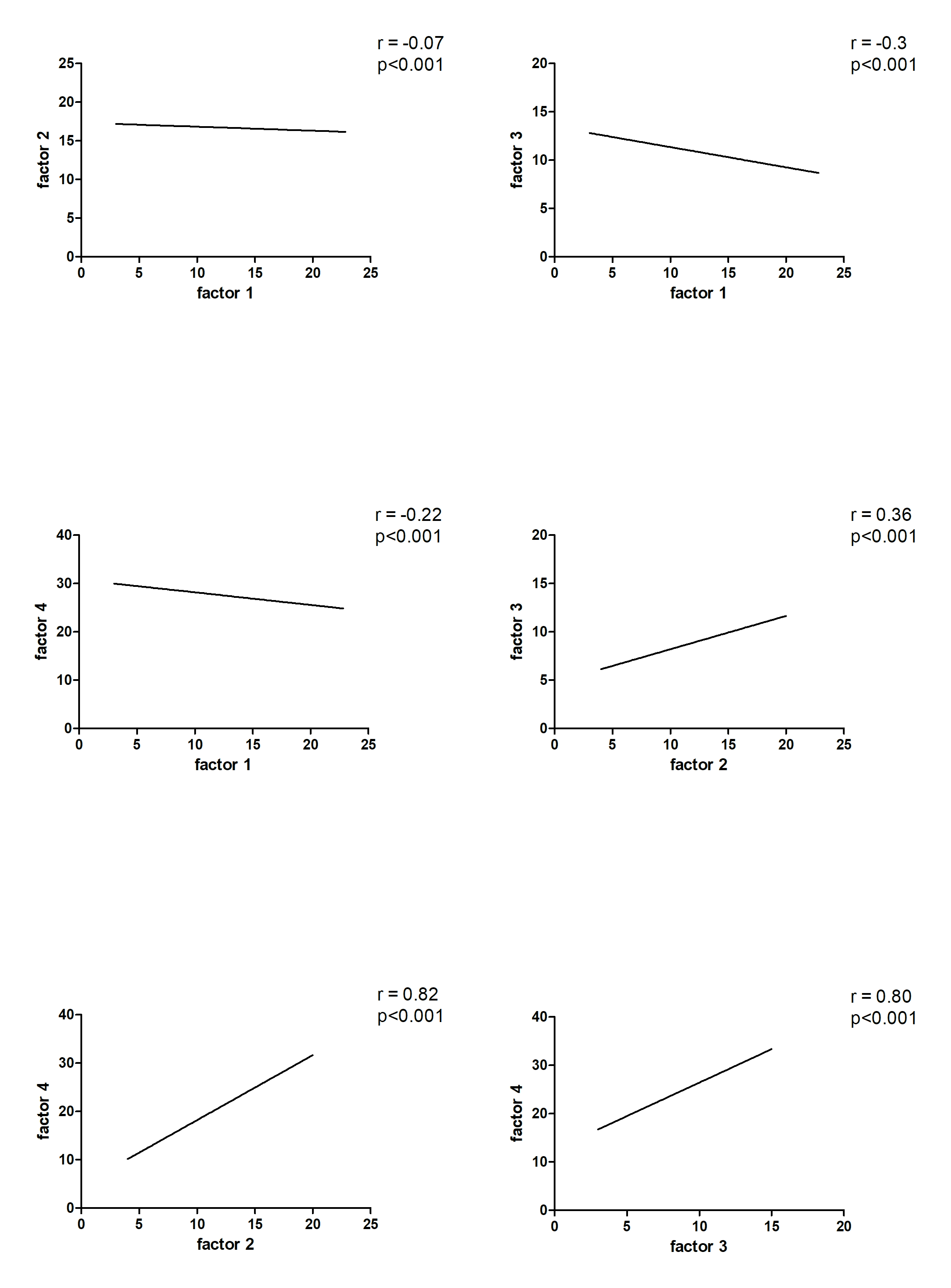

Supplement: Supplementary Figure 1 — Correlation among the four factors: Spearman correlation analysis was performed to assess the correlation among the four factors. In total, six figures were shown to express correlations between each other. Spearman r-value and p-value are shown beside each figure. [file Image_1.TIF]
