# Supplementary material for: Psychological Impact During the First Outbreak of COVID-19 on Frontline Health Care Workers in Shanghai
Source: Front Public Health. 2021 May 17;9:646780. doi: 10.3389/fpubh.2021.646780 (PMC8165161; doi:10.3389/fpubh.2021.646780)
Supplement: Supplementary Table 1 — The score of the four factors with personal characteristics. [file Table_1.DOCX]

**Supplementary table 1. The score of the four factors with personal characteristics**

|  | Factor 1^†^：Anxiety about infection |  |  | Factor 2^‡^：Knowledge about COVID-19 |  |  | Factor 3^§^: Feeling of being protected |  |  | Factor 4^¶^: Attitude towards work |  |  |
| --- | --- | --- | --- | --- | --- | --- | --- | --- | --- | --- | --- | --- |
|  |  |  |  |  |  |  |  |  |  |  |  |  |
|  | Total score | min | max | Total score | min | max | Total score | min | max | Total score | min | max |
|  | （mean±SD） |  |  | （mean±SD） |  |  | (mean±SD) |  |  | (mean±SD) |  |  |
| **Gender** |  |  |  |  |  |  |  |  |  |  |  |  |
| Male | 14.11±3.56 | 5 | 23 | 17.00±2.47 | 4 | 20 | 10.32±2.42 | 3 | 15 | 11.40±2.65 | 3 | 15 |
| Female | 14.19±3.67 | 3 | 23 | 16.55±2.66 | 4 | 20 | 10.50±2.51 | 3 | 15 | 11.27±2.73 | 3 | 15 |
| **Age(years)** |  |  |  |  |  |  |  |  |  |  |  |  |
| ≤25 | 13.58±3.63 | 3 | 23 | 16.11±2.74 | 4 | 20 | 11.29±2.42 | 3 | 15 | 11.84±2.57 | 3 | 15 |
| 26~35 | 14.38±3.71 | 3 | 23 | 16.55±2.68 | 4 | 20 | 10.45±2.56 | 3 | 15 | 11.06±2.75 | 3 | 15 |
| 36~45 | 14.30±3.58 | 4 | 23 | 16.81±2.58 | 4 | 20 | 10.14±2.42 | 3 | 15 | 11.21±2.68 | 3 | 15 |
| ≥46 | 13.87±3.63 | 5 | 23 | 16.72±2.54 | 4 | 20 | 10.64±2.39 | 3 | 15 | 11.60±2.75 | 3 | 15 |
| **Occupation** |  |  |  |  |  |  |  |  |  |  |  |  |
| Physician | 13.81±3.41 | 3 | 23 | 16.72±2.50 | 4 | 20 | 10.00±2.36 | 3 | 15 | 11.09±2.73 | 3 | 15 |
| Nurse | 14.41±3.78 | 3 | 23 | 16.54±2.71 | 4 | 20 | 10.78±2.54 | 3 | 15 | 11.40±2.71 | 3 | 15 |
| **Marital status** |  |  |  |  |  |  |  |  |  |  |  |  |
| Married | 14.35±3.69 | 3 | 23 | 16.75±2.59 | 4 | 20 | 10.37±2.52 | 3 | 15 | 11.26±2.74 | 3 | 15 |
| Unmarried | 13.61±3.46 | 3 | 23 | 16.16±2.75 | 4 | 20 | 10.85±2.41 | 3 | 15 | 11.35±2.66 | 3 | 15 |
| Divorce | 14.92±3.91 | 8 | 23 | 16.90±2.62 | 9 | 20 | 10.21±2.40 | 3 | 15 | 11.35±2.79 | 3 | 15 |
| **Technical title** |  |  |  |  |  |  |  |  |  |  |  |  |
| Junior | 14.20±3.72 | 3 | 23 | 16.40±2.73 | 4 | 20 | 10.75±2.55 | 3 | 15 | 11.23±2.71 | 3 | 15 |
| Middle | 14.32±3.64 | 4 | 23 | 16.79±2.52 | 4 | 20 | 10.18±2.46 | 3 | 15 | 11.24±2.73 | 3 | 15 |
| Senior | 13.45±3.28 | 7 | 23 | 17.02±2.49 | 8 | 20 | 10.20±2.18 | 3 | 15 | 11.81±2.67 | 3 | 15 |
| **Level of Hospital** |  |  |  |  |  |  |  |  |  |  |  |  |
| Tertiary | 14.92±3.69 | 4 | 23 | 16.28±2.66 | 4 | 20 | 10.23±2.55 | 3 | 15 | 11.12±2.85 | 3 | 15 |
| Secondary | 13.88±3.57 | 3 | 23 | 16.92±2.62 | 4 | 20 | 10.89±2.48 | 3 | 15 | 11.42±2.68 | 3 | 15 |
| Primary | 13.94±3.67 | 3 | 23 | 16.46±2.59 | 4 | 20 | 10.11±2.40 | 3 | 15 | 11.23±2.64 | 3 | 15 |
| **Contact history** |  |  |  |  |  |  |  |  |  |  |  |  |
| Yes | 15.01±3.94 | 4 | 23 | 16.87±2.54 | 4 | 20 | 10.41±2.34 | 3 | 15 | 11.35±2.78 | 3 | 15 |
| No | 13.60±3.43 | 3 | 23 | 16.74±2.62 | 4 | 20 | 10.85±2.44 | 3 | 15 | 11.47±2.67 | 3 | 15 |
| Not sure | 15.30±3.72 | 5 | 23 | 16.03±2.70 | 4 | 20 | 9.43±2.49 | 3 | 15 | 10.69±2.74 | 3 | 15 |
