# Supplementary material for: Psychological Impact During the First Outbreak of COVID-19 on Frontline Health Care Workers in Shanghai
Source: Front Public Health. 2021 May 17;9:646780. doi: 10.3389/fpubh.2021.646780 (PMC8165161; doi:10.3389/fpubh.2021.646780)
Supplement: Supplementary Table 2 — Factors and prevalence of psychological problem in different occupation. [file Table_2.DOCX]

Supplementary table 2: Factors and prevalence of psychological problem in different occupation

| **Factors** | **Physician** | **Nurse** | **p** |
| --- | --- | --- | --- |
| Factor 1 (mean±SD)  Anxiety about infection | 13.81±3.41 | 14.41±3.78 | <0.001 |
| Factor 2 (mean±SD) Knowledge about COVID-19 | 16.72±2.50 | 16.54±2.71 | 0.123 |
| Factor 3 (mean±SD) Feeling of being protected | 10.00±2.36 | 10.78±2.54 | 0.011 |
| Factor 4 (mean±SD) Attitude towards work | 11.09±2.73 | 11.40±2.71 | 0.002 |
| GHQ-12（n(%)） | 975(46.8%) | 1584(43.5%) | 0.26 |
